# Authors' Reply

**DOI:** 10.1371/journal.pmed.0020309

**Published:** 2005-09-27

**Authors:** Harri Hemilä, Robert M Douglas

**Affiliations:** **1**University of HelsinkiHelsinkiFinland; **2**Australian National UniversityCanberraAustralia

The responses to our Best Practice article [1] by Hickey and Roberts [2], and by Sardi [3], make the same point, namely, that a recent pharmacokinetic study reported that frequent oral intakes of vitamin C would be necessary to elevate plasma ascorbic acid levels to the point where they believe it would have a pharmacological impact. Both authors suggest that the conclusions of our Cochrane review [4] are flawed because all of the placebo-controlled trials that have been carried out so far have used, for both prophylaxis and therapy, one to three doses per day of vitamin C, ranging from 200 mg daily to as much as 8 g in a single daily dose.

We have not, as our critics imply, concluded that vitamin C, in the doses used in trials reported in the literature, has no effect on the common cold. On the contrary, our evidence indicated that in marathon runners and in those exposed to high physical or cold stress, a substantial prophylactic effect was observed. And in the general population using regular vitamin C prophylaxis, common cold duration was consistently shortened, but the level of shortening was relatively trivial.

We do not consider the vitamin C and the common cold relationship closed. Nor are we persuaded by the arguments of these three critics that frequent, large doses would necessarily result in substantially greater benefits than earlier trials have demonstrated.

We consider that it may be useful to distinguish between (a) prophylactic supplementation for people who are in good health and (b) therapeutic supplementation for people who have an infection. The kidneys reabsorb essentially all vitamin C when the dietary intake is below 60–100 mg/day, and the vitamin C level in leukocytes is saturated by approximately 100 mg/day [5]; in this respect, we doubt that prophylactic supplementation of healthy people, using doses higher than those in the published trials, might be expected to benefit the general healthy population. On the other hand, there is evidence indicating that common cold infection decreases the vitamin C level in leukocytes, suggesting changes in vitamin C metabolism [6], and, in this respect, there seems to be a rationale to study the effects of supplementation on people infected with the common cold using even higher doses.

To this point, the claim that these two letters make has not been reported in properly conducted randomized controlled trials of either therapy or prophylaxis. We look forward to incorporating such trials, when they have been carried out, in future versions of the Cochrane review. Meanwhile, we stand firmly by the conclusions reported in our article.
